# At-Home Care Program for Acute Myeloid Leukemia Induction Phase in Patients Treated with Venetoclax-Based Low-Intensity Regimens

**DOI:** 10.3390/cancers16244274

**Published:** 2024-12-23

**Authors:** Alexandra Martínez-Roca, Carlos Jiménez-Vicente, Beatriz Merchán, Sandra Castaño-Diez, Inés Zugasti, Helena Brillembourg, Álex Bataller, Francesca Guijarro, Albert Cortés-Bullich, Ana Trigueros, Amanda Isabel Pérez-Valencia, Cristina Gallego, Nuria Ballestar, Luis Gerardo Rodríguez-Lobato, Esther Carcelero, Marina Díaz-Beyá, Jordi Esteve, Francesc Fernández-Avilés

**Affiliations:** 1 Department of Hematology, Hospital Clínic Barcelona, 08036 Barcelona, Spain; apmartinez@clinic.cat (A.M.-R.); cajimenez@recerca.clinic.cat (C.J.-V.); bmerchanm@clinic.cat (B.M.); izugasti@clinic.cat (I.Z.); brillembourg@clinic.cat (H.B.); abataller@mdanderson.org (Á.B.); albcortes@clinic.cat (A.C.-B.); atriguero@clinic.cat (A.T.); aiperez@clinic.cat (A.I.P.-V.); cgallego@clinic.cat (C.G.); ballestar@clinic.cat (N.B.); lgrodriguez@clinic.cat (L.G.R.-L.); diazbeya@clinic.cat (M.D.-B.); ffernand@clinic.cat (F.F.-A.); 2 Home Care and Bone Marrow Transplantation Unit, Hospital Clínic Barcelona, 08036 Barcelona, Spain; 3 Medicine, Faculty of Medicine and Health Sciences, University of Barcelona, 08007 Barcelona, Spain; 4 Institut d’Investigacions Biomèdiques August Pi I Sunyer (IDIBAPS), 08036 Barcelona, Spain; scastano@clinic.cat (S.C.-D.); fguijarro@clinic.cat (F.G.); 5 Hemopathology Unit, Pathology Department, Hospital Clínic Barcelona, 08036 Barcelona, Spain; 6 Pharmacy Service, Division of Medicine, Hospital Clínic de Barcelona, 08036 Barcelona, Spain; ecarcele@clinic.cat

**Keywords:** venetoclax, acute myeloid leukemia, at-home management, hypomethylating agents, patient education, azacitidine

## Abstract

**Background:** Even though venetoclax in combination with azacitidine (VenAza) is considered a low-intensity regimen, its patients present a high incidence of cytopenia and infections during the first courses, making the initial management a challenging phase. **Methods:** This difficulty in our center led to the establishment of an At-Home (AH) program for ramp-up and follow-up patients during the VenAza combination induction phase focused on therapy administration, patient and caregiver education, and management of adverse events (AEs). A total of 70 patients with newly diagnosed acute myeloid leukemia (ND-AML) or relapsed/refractory AML (R/R AML) were treated with VenAza from March 2019 to May 2022. We compared outcomes between patients managed with a hospital-based (inpatient) approach and those managed through the AH program. **Results:** Despite most patients experiencing grade 3–4 cytopenias (96.9%), the incidence of serious infections and other AEs was comparable between both groups, with no significant difference in febrile neutropenia (42.3% vs. 27.8%, *p* = 0.38). Overall, the AH cohort demonstrated a significantly lower hospital readmission rate after ramp-up (29.5% vs. 84.6%, *p* = 0.001). Moreover, the inpatient cohort’s admission days were longer than in the AH cohort (13 vs. 8, *p* = 0.28). **Conclusions:** AH management was feasible and safe, leading to better resource use, enhanced patient comfort, and improved treatment compliance. The potential of AH programs for managing low-intensity chemotherapy regimens can reduce hospital admissions and subsequently improve patient and caregiver well-being.

## Simple Summary

Venetoclax combined with azacitidine (VenAza) in patients with acute myeloid leukemia (AML) presents a high incidence of cytopenias and infections during initial treatment cycles, making early management challenging. To address this, our center implemented an At-Home (AH) program during the VenAza induction phase, focusing on therapy administration, patient and caregiver education, and adverse events (AEs) management. From March 2019 to May 2022, 75 patients with newly diagnosed or relapsed/refractory AML were treated with VenAza, with the experiment comparing outcomes between a hospital-based (inpatient) cohort of 24 patients initially admitted for treatment administration and an AH cohort (*n* = 44). Although most patients experienced grade 3–4 cytopenia (96.9%), the incidence of serious infections and other AEs was similar between groups. The AH cohort had a significantly lower hospital readmission rate after ramp-up (29.5% vs. 84.6%, *p* = 0.0001) and shorter hospital stays (8 vs. 13 days, *p* = 0.28). AH management proved to be safe and effective, optimizing resource use and improving patient and caregiver well-being.

## 1. Introduction

Since the results of the VIALE-A trial showed a beneficial outcome of the experimental arm, venetoclax in combination with azacitidine (VenAza) has become the standard of care as frontline treatment in unfit patients newly diagnosed with acute myeloid leukemia (ND-AML) [1,2]. VenAza has also demonstrated itself to be a useful therapeutical option in relapsed/refractory (R/R AML), including patients who receive this treatment scheme as preemptive therapy when presenting a molecular relapse [3,4,5,6]. 

Despite being a low-intensity regimen, VenAza induces deep and prolonged cytopenias [1] in most patients at a relatively high incidence during the first courses, as well as infections, making the initial management until the achievement of response and hematological recovery a challenging phase.

Initially, all patients treated at our institution were admitted to the hospital for the ramp-up phase to prevent tumor lysis syndrome (TLS) mainly observed in other hematologic malignancies where venetoclax had already been used [7]. Since our initial experience with VenAza showed a low toxicity profile and the virtual absence of TLS during ramp-up, it prompted us to develop an At-Home (AH) program in February 2020, focusing on therapy administration, patient and caregiver education, as well as management of hematological and non-hematological toxicities associated with this regimen during the induction phase (1–2 courses), which is, in our experience, the phase of maximum patient vulnerability, until a hematological response is observed.

Herein we present our unicentric experience of the combination of VenAza in patients with ND-AML and R/R AML, focusing on the impact of the AH program for initial management in comparison with the cohort of patients receiving a hospital-based standard initial management. The advent of multiple novel low-intensity combinations, including triplet combinations with targeted agents (e.g., FLT3, IDH, and Menin inhibitors), highlights the need to develop AH care programs for managing the initial phase of these low-intensity regimens.

## 2. Patients and Methods

### 2.1. Patient Inclusion and Supportive Measures

Our main goal was to evaluate the impact of an AH program for patients treated with VenAza compared to a control group of patients treated with the same regimen and conventional management, including admission for initial ramp-up.

We performed a retrospective, unicentric analysis of all adult patients older than 18 years who were consecutively treated with VenAza at our institution, either for ND-AML in unfit patients or for R/R AML, including those presenting molecular failure (persistence or reappearance of measurable residual disease (MRD)) [8]. Therefore, the study comprised two cohorts accrued from March 2019 to May 2022, comprising an initial cohort of conventionally treated patients and a second cohort consisting of patients included in the AH program. 

An initial ramp-up of venetoclax and adequate tumor TLS prophylaxis was given in both inpatient and AH according to our institution’s protocol. In the inpatient cohort, patients were admitted for completing the ramp-up (5 days), and, at discharge, they were followed up in our outpatient clinic, contrary to the AH program, where ramp-up and follow-up were done outpatient or at home by the Home Care Unit (HCU). Cytopenia per se was not a criterion to delay the discharge of patients admitted for the ramp-up.

The Ethics Committee of the Hospital Clínic of Barcelona approved the study following the Declaration of Helsinki. 

AML and myeloid-related diseases were classified according to the ICC 2022 classification [9], and disease risk stratification was assessed according to the 2022 European LeukemiaNET (ELN 2022) [10]. ELN risk was based on genetic testing at the time of inclusion to the study for both ND-AML and R/R AML patients. Adverse events were determined according to the Common Terminology Criteria for Adverse Events (CTCAE), Version 5.0, November 2017.

### 2.2. At-Home Management Program

**Figure 1:**
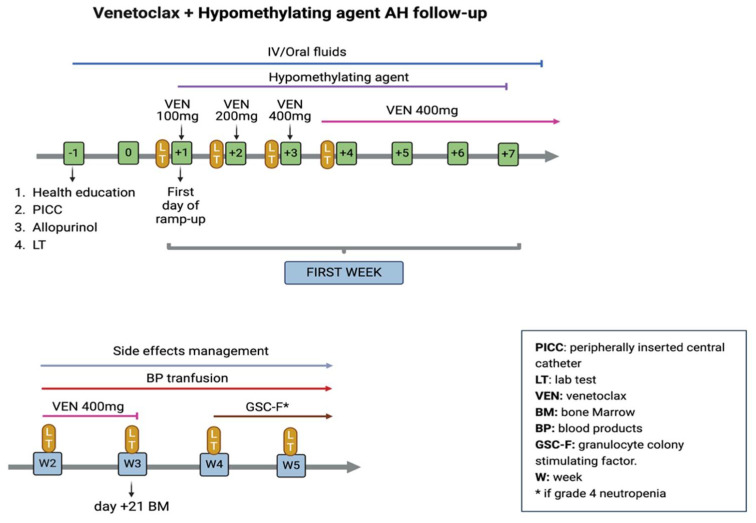
Ramp-up of venetoclax and prophylactic measurements to prevent tumor lysis syndrome during the first days of cycle 1 of venetoclax in combination with hypomethylating agents, and co-secutive follow-up. Created with BioRender.com. Accessed on 16 December 2024.

In February 2020, a specific AH program for patients receiving VenAza was implemented, as well as the conception of a specific protocol for adapting the ramp-up AH and early follow-up period. Patients were followed for around 60 days (two cycles) before achieving a hematological response to prevent and manage adverse events (AEs). The medical inclusion criteria to take part in the VenAza AH program included a total white blood cell (WBC) count <25 × 10^9^/L, an absence of active infections or severe organ comorbidities (i.e., cardiac, pulmonary, renal, hepatic), and an absence of neurological or psychiatric disorders. Furthermore, logistical inclusion criteria were also required: residence at a maximum of 60 min of traveling distance from the hospital and, in case of a longer distance, capacity to travel with a caregiver to our AH support unit (Supplemental Figure S1). Caregiver availability and willingness to participate in this program were also required. Before therapy initiation, a comprehensive medical evaluation was performed by a physician and a liaison nurse from the HCU unit. Previous medical history, potential drug interactions, and comorbidities were specifically assessed. Laboratory tests were performed, as well as a rectal swab to detect previous multiresistant bacterial colonization. Finally, extensive health education on side effects and HCU procedures was provided to both the caregiver and the patient. After ramp-up, a minimum number of visits, lab tests (once weekly), and phone visits by our nursing staff (twice weekly) was established. This schedule was intensified on a 7-day basis, adapted to transfusion demand or surveillance of an emergent AE. This continuous patient follow-up allowed us to detect emerging AEs, review patient compliance with medication, offer continuing education in AEs detection, and resolve patient inquiries related to the therapy. A graphic description of the AH ramp-up and follow-up is presented in Figure 1.

For AEs presenting after therapy initiation (e.g., fever, bleeding), patients were advised to attend our specific 24-h support unit (SU), where they were rapidly evaluated by a hematologist, allowing us to institute a prompt intervention and prevent hospital admission for most patients. Our support unit consists of a hospital bed located in one of our hematological wards. Nurses in the HCU are available from 8:00 a.m. to 10:00 p.m. in two shifts for 7 days a week. 

Medical care during the remaining period (i.e., from 10 p.m. to 8 a.m.) was provided directly by an on-call hematologist. The complete AH program protocol is detailed in the Supplementary Material.

## 3. Statistical Analysis

All baseline characteristics and clinical courses during treatment were retrospectively collected from the electronic records in our center. During follow-up, patients were censored either at refractoriness to treatment, progressive disease, or loss of follow-up. 

A descriptive statistical analysis of the main characteristics of all patients included in the study was performed. The median and range were used for continuous variables, and frequency and percentage were used for categorical variables. A Fisher’s exact test or χ^2^ test were used for univariate analysis in categorical variables, and a Wilcoxon rank sum or *t*-test were used for continuous variables. The main endpoints of interest for comparison of both patient cohorts (i.e., inpatient and AH cohorts) were the re-admission rate (hospital stay of more than 24 h), days of hospitalization, and incidence of AEs.

All *p* values were two-sided with statistical significance evaluated at the 0.05 alpha level. All statistical analyses were performed with R statistics version 4.0.2 (R core Team, R Foundation for Statistical Computing, Vienna, Austria).

## 4. Results

From March 2019 to May 2022, 70 patients diagnosed with AML and two additional patients with T-lymphoid/myeloid mixed-phenotype acute leukemia were considered eligible for VenAza treatment. Of these 70 patients, 28 were ND-AML. In contrast, the remainder (42) received VenAza for R/R AML, of which seven presented a molecular relapse after an initial response to standard chemotherapy. Interestingly, 25 patients (33.3%) out of the R/R AML patients had received hypomethylating agents in previous lines. All baseline characteristics of the study population are displayed in Table 1.

**Figure 2:**
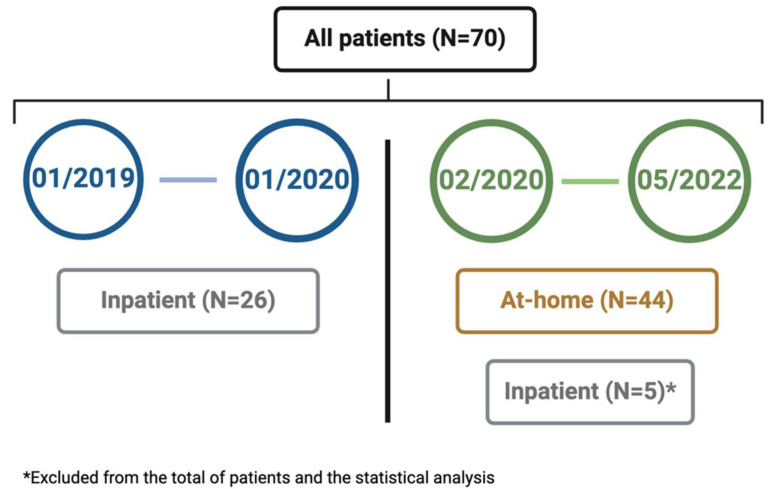
Inpatient vs. AH distribution of patients treated with VenHMA throughout time. Created with BioRender.com, accessed on 16 December 2024.

Twenty-six patients were initially treated for ramp-up as inpatients before the existence of the AH program. Since the beginning of the AH program (February 2020), five ND-AML patients were not included in the AH for the ramp-up due to concomitant complications at diagnosis, including two patients with hyperleukocytosis (WBC count > 25 × 10^9^/L), one of them with concomitant sepsis. The remaining three patients presented with severe infectious complications (pneumonia due to SARS-CoV-2, sepsis with positive cultures for coagulase-negative staphylococci, and a possible fungal infection). These patients were excluded from the analysis (Figure 2). Forty-four patients in total were included in the AH program.

A comparison of the main characteristics between both cohorts is also provided in Table 1. Briefly, patients in the AH program were older (74 vs. 65, *p* = 0.001), presented a better performance status (PS) (ECOG 0–1, 88.6% vs. 57.7% *p* = 0.007), and presented with a higher neutrophil (1.3 vs. 0.46 ANC ×10^9^/L, *p* = 0.001) and platelet (PLT) count at baseline (50 vs. 36 PLT ×10^9^/L, *p* = 0.001). Most patients of both cohorts were diagnosed with an adverse risk AML (according to ELN 2022).

### 4.1. Outcome of Patients

The overall response rate after cycle 2 (C2) in our series was 46.7% in ND-AML patients and 35% in R/R AML patients. The median overall survival of the whole cohort was 8 months, or 18.7 months (95% CI 10.3–NA) in ND-AML patients and 6.91 months (95% CI 4.97–9.44) in R/R AML patients.

### 4.2. Resource Use, Adverse Events, and Need for Admission in the At-Home Cohort

A total of 176 planned AH visits were made, either for treatment administration or to collect blood tests, representing an average of 13 visits per AH patient. In addition, 119 programmed visits (an average of 9 per patient) were done in our SU, either for blood transfusions or to administrate subcutaneous/intravenous (SC/IV) treatment (e.g., Hypometilants (HMA), antibiotics (ATB)). Forty-one extra visits were also recorded for emerging AEs in our SU, representing an average of 1.8 additional visits per patient (range 1–5). In 29 of these visits (70%), a 24 h stay for observation was required, and 19 (43%) patients were able to receive at-home/outpatient parenteral ATB treatment following this short observation stay, accounting for a total of 177 days (median of 9.2 days per patient) without the need for hospital admission.

Sixty-six patients (94.3%) presented any grade of neutropenia, (100% in the inpatient vs. 88.9% in the AH cohort, *p* = <0.001), with 59 (85.3%) of all the patients (80.8% vs. 86.3% respectively) developing grade 4 neutropenia during treatment. Any grade of thrombocytopenia was observed in 80.8% and 86.3.% of inpatient and AH cohorts, respectively (*p* = 0.03), although acute bleeding (grade 1) was observed only in a minor proportion of cases: 7.7% vs. 2.8%, *p* = 0.56, respectively. Grade 3–4 anemia was observed in 38 patients (54.3%).

Thirty-nine (57.7%) of all patients included presented any documented infection during treatment. No statistically significant differences were seen between both cohorts regarding infection type. Febrile neutropenia after therapy initiation was present in 26 (37.1%) patients (34.6% inpatient vs. 27.8% AH, *p* = 0.38), proven bacterial infection in 18 (25.7%, 31% vs. 19.4%, *p* = 0.25 respectively), proven viral infection in 6 (8.6%, 8.3% vs. 11.1%, *p* = 0.22 respectively)—including 2 patients diagnosed with a SARS-CoV-2 infection during the first two cycles in the AH cohort—and probable/proven fungal infections in 7 (10%) patients, 15.4% vs. 8.3%, *p* = 0.43, respectively. Among bacterial infections, two patients in the inpatient subgroup and one in the AH group presented sepsis requiring hospital readmission.

Three cases of deep vein thrombosis (two and one from the inpatient and AH cohorts, respectively) were documented during treatment. Cardiac events were observed in three (4.3%) patients: one case of stent thrombosis in the inpatient cohort, and two atrial fibrillations, one in each cohort. No episodes of TLS were observed in any of the subgroups. Two patients, one in each cohort, died within the first 30 days (one for septic shock in the inpatient group and one due to SARS-CoV-2 infection in the AH cohort). Four patients were admitted to the intensive care unit (ICU) in the inpatient cohort and one in the AH (12.9% vs. 2.2%, *p* = 0.10). All adverse events are detailed in Table 2.

The impact of AH management was seen in a significantly lower readmission rate after ramp-up (84.6% vs. 29.5%, *p* = 0.001). Moreover, to explore the confounding factors associated with this, we performed a logistic regression analysis (Supplemental Table S1) that confirmed that the AH program was significantly associated with a lower readmission rate. Also, the inpatient cohort’s admission days were considerably longer than in the AH cohort (14 vs. 8, *p* = 0.28) (Supplemental Figure S2).

## 5. Discussion

The present study summarizes the main results of a pioneer AH program designed in our center for the initial management of patients treated with VenAza during the first two cycles of treatment (approximately 60 days), the phase with a higher incidence of severe AEs. Interestingly, this analysis proved that the AH is feasible, safe, and results in a low hospital readmission rate. Our experience in at-home management of highly complex procedures started more than 24 years ago in patients undergoing autologous hematopoietic stem cell transplantation (ASCT), mostly diagnosed with multiple myeloma and lymphoma, which shows the feasibility and safety of at-home management, as well as reproducibility in other hematological centers [11,12,13,14,15]. 

At-home management has subsequently been extended to other procedures such as the aplastic phase after consolidation therapy in AML, consolidation therapy with arsenic trioxide (ATO) for acute promyelocytic leukemia, a pilot program in selected patients for allogeneic hematopoietic stem cell transplant (Allo-SCT), and, more recently, a program for outpatient CAR-T cell product infusion and at-home follow-up. In this context, we identified the ramp-up phase for patients diagnosed with chronic lymphocytic leukemia (CLL) and AML receiving venetoclax-based therapy as another potential target that can benefit from an AH program.

Despite the wide use of the VenAza combination in chemotherapy-ineligible AML patients, hospital admission for initial ramp-up management is highly recommended [16]. Initially, the TLS risk was expected to be higher based on previous observation of patients with CLL treated with venetoclax, although the TLS rate has been proven to be lower in patients with AML [17]. Two preliminary studies by C. Papayannidis et al. [18] in Italy and Pelcovits et al. [19] in the United States described the first experience of an outpatient ramp-up and follow-up program during the first 28 days of treatment. In both studies, patients always had to attend the hospital for visits, contrary to our program, where patients are mostly visited at home, and, as explained before, they benefit from constant follow-up and education, as well as prompt management of AEs thanks to our 24/7 SU and the rapid evaluation of an on-call hematologist. The two studies also report a low TLS incidence of <5%. In our experience, no cases of laboratory or clinical TLS were observed in either the AH or the inpatient group, which is probably attributable to a combination of specific selection criteria for patients admitted to the AH program, TLS prophylactic measures implemented before therapy initiation, and daily monitoring of TLS parameters. In the Italian study, they also report a low incidence of readmission in the outpatient group, as shown by our work. A similar experience has also been published recently, showing the feasibility, safety, and reproducibility of outpatient care [20]. Moreover, an alternative to hospital admission for VenAza ramp-up could result in significant economic saving. A positive cost reduction in favor of the outpatient ASCT model, for example, ranging from 19.32% to a 46.48% reduction, is an excellent reflection of cost saving with outpatient/at-home management of normally inpatient procedures [21], as well as an optimization of hospital resources in the context of lack of hospital beds in most hospitals.

Our previous experience administering HMA at home since 2018, as published by other centers [22], was the starting point to establish a protocol for performing ramp-up and AEs follow-up in our AH unit. 

Regarding hematological AEs, myelotoxicity was the most common adverse event in both the hospitalized and AH cohorts. Interestingly, most patients presented with grade 3 or 4 neutropenia or thrombocytopenia before VenAza began. Starting antibacterial and antifungal prophylaxis when HMA agents are combined with venetoclax are suggested, especially during profound neutropenia. Antifungal prophylaxis with posaconazole is recommended for dose adjustment due to the inhibition of the CYP3A cytochrome [23], even though the clinical efficacy of the reduced dosage of venetoclax is still unknown [18].

In our cohort, patients were started on antimicrobial prophylaxis with fluoroquinolones and antifungal prophylaxis with isavuconazole to reduce interaction with venetoclax when severe neutropenia was present. With this attitude, 8.3% of probable/proven fungal infections were present in our AH group, which is lower than previous reports [24]. Nevertheless, low rates of IFI using VenAza have been reported [23,24]. Thus, we believe that antifungal prophylaxis is essential because the prediction of neutropenia duration in this type of patient is unknown, and, in most cases, it correlates to a morphological response of the disease [24]. Dose reduction of VenAza after achieving a response is required in most patients and is essential to avoiding prolonged cytopenia-related adverse events, as recently published by our group [25].

Although no differences were seen in febrile neutropenia in both groups (42.3% vs. 27.8%, *p* = 0.38), a significantly lower readmission rate after ramp-up (84.6% vs. 29.5% *p* = 0.001) in the AH-managed patients was observed, as well as a trend of a lower number of admission days (13 vs. 8, *p* = 0.28). Patients in the AH cohort benefited from careful daily management and continuous patient and caregiver education, which translated into better follow-up of treatment, with a substantial subjective improvement in the well-being of patients and caregivers. To evaluate health-related quality of life in our AH program, we recently started recollection of data assessing the quality of life at different points in time to objectively confirm the advantages of AH management in role, emotional, and social function [26].

We presume that the low rate of hospital readmission could be explained, in part, by the close, pre-scheduled patient monitoring program, which included medical visits, blood tests during the aplastic phase, and regular phone visits by a trained nurse team, and which could be adapted to transfusion demand and emergence of AEs. Also, patients presenting an unscheduled AE potentially requiring hospital admission could be attended in the SU and, and once clinical stability was achieved, they were able to return to the AH program to complete treatment (e.g., IV ATB) under the HCU in more than 40% of instances. In a cohort that is mostly composed of patients with a median age of 71 years and concomitant co-morbidities—and in some cases, a lower physical mobility—this program allows us to maintain a lower rate of hospital visits.

In the matter of cardiac events, we observed only 4.3% in both groups in comparison with previous reports [27]. A meticulous review of medical history, comorbidities, and potential pharmacological interactions with venetoclax was performed before therapy initiation, and in patients with a history of cardiac events, an echocardiogram was performed. 

Even though a cost analysis of our program has not been performed, we can speculate that AH ramp-up could be a less expensive option, as demonstrated before with the AH ASCT and AH Allo-SCT experience [28]. Also, our AH strategy reduces hospital admissions and may reduce health care expenses while improving patient comfort and encouraging patient and caregiver empowerment.

This study has some limitations as a retrospective, single-center-based study, and requires corroboration with larger cohorts. Also, patients included in the AH program for MRD relapse presented with better performance status (PS) laboratory values, as well as lower transfusion dependency, but we did not observe any differences between ND-AML and R/R AML patients in both cohorts. Regarding the limitations of our AH program, in some cases, patients are not close to our center (>60 min), so they must attend the hospital to perform blood tests and to receive blood transfusions. However, they also benefit from our daily follow-up and rapid management of AEs.

## 6. Conclusions

In the subset of patients qualified as “unfit” for high-intensity treatment, the VenAza combination presents as an optimal option for AML treatment, allowing a comfortable administration as an outpatient or even, as our experience shows, at home. In addition, our AH program can help to improve patient follow-up and presents as a feasible and safe option with a low readmission rate that offers a wide range of benefits, such as the optimization of health resources and an increase in the comfort and well-being of patients and their caregivers. While VenAza is considered a “low-intensity” regimen, a close follow-up is mandatory to detect neutropenia, an early complication related to the therapy.

## Figures and Tables

**Table 1:** Baseline characteristics of all patients.

Characteristics	All Patients (*n* = 70)	Inpatient (*n* = 26)	At-Home (*n* = 44)	*p*
**Age, median (range)**	71 (23–85)	65 (29–80)	74 (23–85)	0.001
**Sex**				1
Female	26 (37.7)	10 (38.5)	16 (36.4)	
**ECOG performance status**				0.007
0–1	54 (77.1)	15 (57.7)	39 (88.6)	
**ELN** **2022 genetic** risk *§				0.79
Favorable	9 (12.3)	4 (15.4)	5 (11.4)	
Intermediate	13 (17.8)	4 (15.4)	8 (18.2)	
Adverse	51 (68.5)	18 (69.3)	30 (68.2)	
**Previous treatment received**				
Frontline therapy	28 (37.3)	5 (19.3)	18 (40.9)	0.06
Intensive chemotherapy	31 (41.3)	16 (61.5)	15 (34.1)	0.20
Allogeneic hematopoietic stem cell transplant	14 (18.7)	7 (34.6)	7 (15.9)	0.66
Hypomethylating agent	25 (33.3)	9 (29)	16 (36.4)	0.80
**Number of previous treatments received, median (range)**	1 (0–4)	1 (1–4)	1 (0–3)	0.71
**CBC count, median (range)**				
ANC (×10^9^/L)	1.1 (0–12.3)	0.46 (0–12.3)	1.3 (0–12)	0.001
Hemoglobin (g/dL)	9.3 (6.2–14.7)	8.5 (6.2–13.2)	9.2 (7.1–14.7)	0.001
Platelets (×10^9^/L)	55 (7–421)	36 (7–421)	50 (7–269)	0.001
**Circulating blast (range)**	3 (0–98)	14 (0–98)	0.5 (0–86)	0.007

ECOG: Eastern Conference Oncology Group. CBC: complete blood cell count, ANC: absolute neutrophil count. * Risk stratification according to the European LeukemiaNET 2022 recommendations. § An Oncomine Myeloid Research Assay next-generation sequencing panel was performed in all patients at diagnosis or before starting treatment in the R/R AML patients.

**Table 2:** Adverse events of all patients and divided by patients who started ramp-up through a hospital admission and those who received at-home treatment.

Adverse Events During First Two Cycles	All Patients (*n* = 70)	Inpatient (*n* = 26)	At-Home (*n* = 44)	*p*
**Any Adverse Event, no. (%)**	68 (97.1)	26 (100)	42 (97.2)	0.52
**Neutropenia, no. (%)**	66 (94.3)	26 (100)	32 (88.9)	<0.001
Grade 3	7 (9.3)	5 (19.2)	2 (4.5)	
Grade 4	59 (85.3)	21 (80.8)	38 (86.3)	
**Thrombocytopenia, no. (%)**	66 (94.3)	24 (86.2)	33 (90.9)	0.03
Grade 1–3	14 (20)	1 (3.8)	13 (5.6)	
Grade 4	52 (74.3)	23 (88.5)	19 (63.6)	
**Anemia, no. (%)**	63 (90)	25 (96.7)	38 (86.3)	0.39
Grade 2	25 (35.7)	13 (48.3)	12 (27.3)	
Grade 3–4	38 (54.3)	12 (53.3)	26 (59)	
**Infections, no. (%)**	39 (55.7)	17 (65.4)	22 (50)	0.35
Febrile neutropenia	26 (37.1)	11 (42.3)	13 (27.8)	0.38
Bacterial infection	18 (25.7)	9 (34.6)	8 (19.4)	0.25
Viral infection	6 (8.6)	3 (8.3)	2 (11.1)	0.22
COVID-19	2 (2.9)	0 (0)	2 (8.3)	0.5
Fungal infection	7 (10)	4 (15.4)	3 (8.3)	0.43
**Acute bleeding, no. (%)**	3 (4.3)	2 (7.7)	1 (2.8)	0.56
**Cardiac events, no. (%)**	3 (4.3)	2 (7.7)	1 (2.8)	0.56
**Sepsis, no. (%)**	3 (4.3)	2 (7.7)	1 (2.8)	0.56
**Deep vein thrombosis, no. (%)**	3 (4.3)	2 (7.7)	1 (2.8)	0.56
**Hospital admission after ramp-up, no. (%)**	35 (50)	22 (84.6)	13 (29.5)	0.001
Hospitalization days after ramp-up, median (range) *	12 (1–63)	13 (1–63)	8 (1–39)	0.28
**ICU admission**	5 (7.1)	4 (12.9)	1 (2.2)	0.10
**Mortality within first 30 days of treatment, no. (%)**	2 (2.9)	1 (3.2)	1 (2.8)	1

* Hospitalization days were only measured in patients who required admission.

## Data Availability

The original contributions presented in this study are included in the article/Supplementary Material. Further inquiries can be directed to the corresponding author.

## Supplementary Materials

The following supporting information can be downloaded at: https://www.mdpi.com/article/10.3390/cancers16244274/s1, At-home management proposed protocol, Supplemental Figure S1: Selection criteria for the VenAza AH program, categorized into medical and logistic inclusion criteria; Supplemental Table S1: Logistic Regression Analysis of Factors Influencing Readmission. Supplemental Figure S2: Comparison of (A) hospital admission after ramp-up and (B) Intensive care unit (ICU) admission in both cohorts. Length of days of hospitalization after ramp-up (C).
