# Supplementary material for: At-Home Care Program for Acute Myeloid Leukemia Induction Phase in Patients Treated with Venetoclax-Based Low-Intensity Regimens
Source: Cancers (Basel). 2024 Dec 23;16(24):4274. doi: 10.3390/cancers16244274 (PMC11674946; doi:10.3390/cancers16244274)
Supplement: Supplementary file 1 [file cancers-16-04274-s001.zip › cancers-3323393-supplementary.pdf]

Supplementary

AH proposed protocol.

1. *Initial evaluation*

Before therapy started, the selection criteria for the VenAza AH program were evaluated (figure S1). A medical assessment was performed by a Home Care Unit (HCU) physician and an HCU liaison nurse. Previous medical history, potential drug interactions that required venetoclax dose reduction, and comorbidities were evaluated (Especially cardiological and renal), also, patient distance to the center, and number and type of caregivers. Laboratory tests to detect hyperuricemia and cytopenia before the treatment were done, as well as a rectal swab to detect previous bacterial colonization. Also, extensive health education was given that included, treatment scheme, side effects, as well as education in adverse events (AEs) prevention. We also provided both caregivers and patients with verbal and printed information on HCU procedures, phone numbers for contacting during working hours and after hours, and a protocol in case of the appearance of AEs (contacting by phone the treating team during working hours and an on-call hematologist after hours), in case of requiring urgent evaluation, patients were advice to attend to our support unit for prompt evaluation, and in case of so, starting parenteral antibiotic. Evaluation of caregiver burden was also estimated, and recommendations to prevent and improve in case of so were given.

2. *Ramp-up phase:*

At the start of the program, intravenous hydration with a portable pump using a peripheral intravenous central catheter (PICC) was started 24 hours before therapy began, as well as agents to reduce uric acid (allopurinol), patients were advised to maintain oral hydration as well. In February 2022 we decided (due to the absence of tumor lysis syndrome (TLS) in the group of patients treated so far) that only patients presenting with blast in peripheral blood, hyperleukocytosis, and/or previous history of chronic renal impairment or hyperuricemia to be started on an IV pump during ramp-up, if the patient didn't fulfill these criteria, oral hydration was selected. We also restricted PICC placement, only for patients with a high requirement of blood products (more than 2 a week) involving frequent blood draws, and those with poor vein access.

Daily morning visits were performed by trained nurses who reviewed vital signs, and collected laboratory tests, assessed therapeutic compliance, reviewed fluid balance and weight, and replaced portable pumps. Patients were started on venetoclax in escalation doses of 100 mg on day 1, 200 mg on day 2, and 400 mg on day 3 of the cycle, they were advised to take venetoclax after dinner, and only after the confirmation from our team to do so after reviewing the patient laboratory test that was performed daily in the morning (12 hours after VEN dose). Dose reduction was done in patients who were also receiving CYP3A4 inhibitors such as azole antifungals (50% reduction in case of potent CYP3A4 inhibitors and 75% reduction dose in case of strong CYP3A4 inhibitors). Hypomethylating agent (Azacitidine or Decitabine) was started concomitantly with venetoclax on day 1 of the cycle. After achieving the maximum dose of venetoclax and

verifying no analytical signs of TLS, intravenous hydration (in case of so) ceased at day +4 of the cycle (figure 2).

3. *Subsequent therapy:*

Patients were followed throughout the whole cycle by the HCU team. A minimum number of visits was established (at least once weekly for lab tests to detect neutropenia appearance or the necessity of blood products). Patient visit could also be adapted to his/her transfusion support, this was also accompanied by at least two weekly phone visits by our nursing staff (or daily phone visits in case of need) including weekends. Platelet transfusion was administered AH, while blood cell transfusion was provided at the hospital due to our transfusion policy.

Antimicrobial prophylaxis with quinolones and antifungal prophylaxis with isavuconazole (mild inhibitor of CYP3A4) was started as soon as patients presented grade 3 neutropenia (<0,5 $x10^9$ /L). Prophylaxis was individualized in patients who presented previous colonization in rectal swabs.

Bone marrow aspirate was performed at day +21 of the cycle for evaluation of response. In patients who achieved a morphological response in bone marrow, meaning <5% blasts regardless of hematological recovery (CR/CRi/MLFS) granulocyte stimulant was started to shorten the duration of the neutropenia.

Patients who achieved transfusion independence and no AEs (except cytopenia due to therapy), were discharged from the HCU to continue treatment with one of our leukemia physicians as outpatient visits.

In the early phase of the program, many logistical questions arose, since it coincided with the first wave of SARS-CoV-2 and a general lockdown affecting Spain, the HCU unit concentrated on only performing At-home autologous stem cell transplantation, stopping the remaining activity including the AH administration of VenHMA until June 2020. New measures were implemented to improve patients, caregivers, and health workers safety, resulting in a low rate of SARS-CoV-2 in our patients, with no deaths related to the virus after implementing new measures.

Supplemental Figure S1: Selection criteria for the VenAza AH program, categorized into medical and logistic inclusion criteria.

Selection Criteria for VenAza At-Home Program

Medical Inclusion Criteria:

a. Total white blood cell (WBC) count < 25 × 10°/L
b. Absence of active infections
c. No severe organ comorbidities:

- Cardiac
- Pulmonary
- Renal
- Hepatic

d. No neurological or psychiatric disorder

Logistic Inclusion Criteria:

a. Residence within 60 minutes of traveling distance from the hospital
b. For longer distances, the capacity to travel with a caregiver to the Home Care support unit
c. Caregiver availability and willingness to participate

Supplemental Table S1: Logistic Regression Analysis of Factors Influencing Readmission

	Estimator	St.Error	Z-Value	97,5% CI	*P*

**Intercept**	0.13	1.03	0.12	[-1.96, 2.14]	0.89
**Age>71**	0.17	0.29	0.29	[-0.95, 1.38]	0.76
**ECOG PS**	0.44	0.58	0.58	[-1.05, 1.99]	0.55
**AH program**	-1.89	-2.89	-2.89	[-3.27, -0.67]	**0.0038**
**Neutropenia grade 4**	-0.02	-0.05	-0.05	[-1.16 -1.14]	0.95
**Thrombocytopenia grade 4**	0.81	0.85	0.85	[-0.96 – 2.85]	0.39

CI, confidence interval; ECOG PS, Eastern Cooperative Oncology Group performance status; St.Error. Standard Error

Supplemental Figure S2: Comparison of (A) hospital admission after ramp-up and (B) ICU admission in both cohorts. Length of days of hospitalization after ramp-up (C).

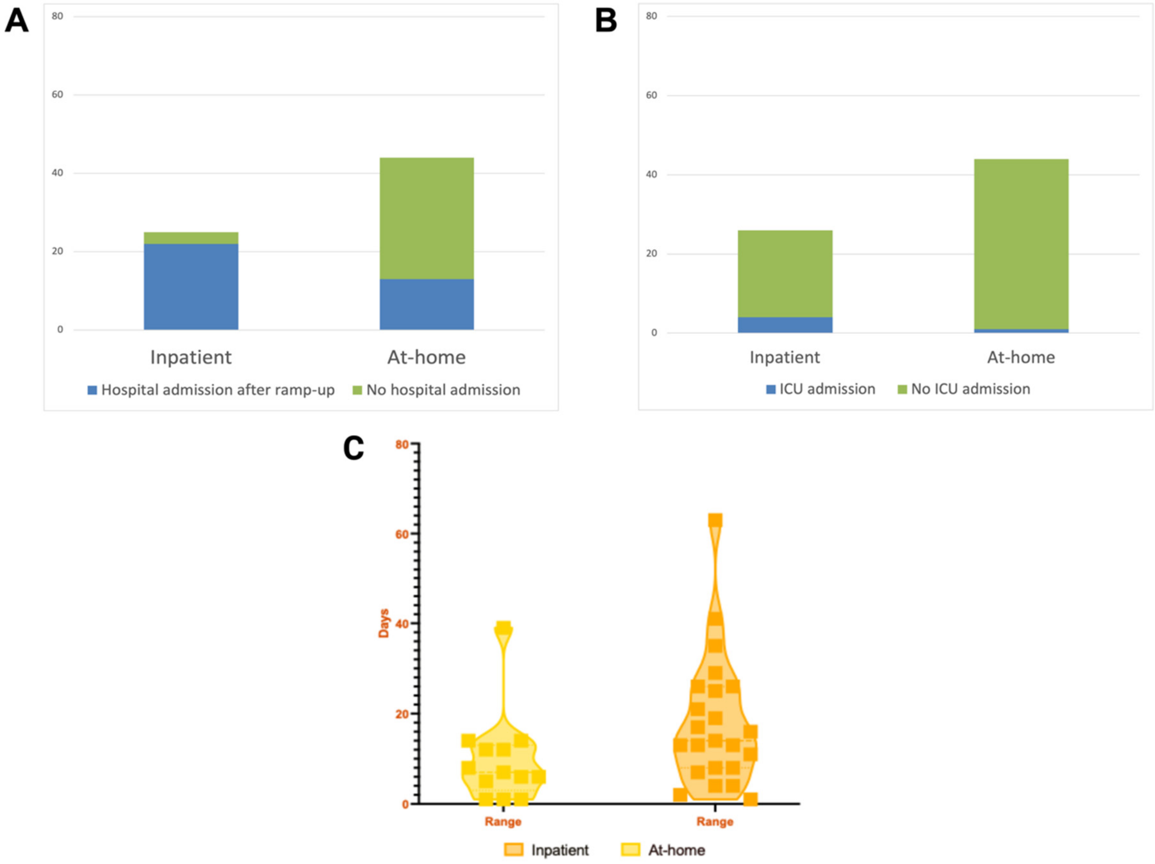

A
80
60
40
20
0
Inpatient
At-home
Hospital admission after ramp-up
No hospital admission
B
80
60
40
20
0
Inpatient
At-home
ICU admission
No ICU admission
C
80
60
40
20
0
Days
Range
Range
Inpatient
At-home
